# Volatile anaesthetics for analgesia and sedation in patients after abdominal surgery in ICU

**DOI:** 10.1186/2197-425X-3-S1-A329

**Published:** 2015-10-01

**Authors:** VI Potievskaya, IV Molchanov, TV Krivenko

**Affiliations:** Russian Medical Academy of Postgraduate Education, Anaesthesiology and Intensive Care, Moscow, Russian Federation

## Introduction

It is known that volatile agents are efficient and safe in general anaesthesia and may be used in ICU as alternative to intravenous medications [1]. Neverhteless we lack information about special features of analgesia and sedation and haemodynamic changes during action of inhalational anaesthetics in patients after surgery.

## Objectives

The objective of this prospective study was to evaluate efficacy of analgesia and sedation with volatile anaesthetic sevoflurane and its influence on haemodynamics in mechanically ventilated patients in ICU.

## Methods

37 patients on prolonged mechanical ventilation (20 males, 17 females) after abdominal surgery were followed up in ICU. The sedation level was evaluated with RASS scale and auditory evoked potentials (AEP), analgesia - with the analgesia/nociceptive index (ANI), based on spectral analysis of RR intervals [2], hemodynamic parameters - by impedance plethysmography. Sedation was performed by AnaConDa (anaesthetic conserving device) with sevoflurane. Mean duration of sedation was 5 hours. Statistical analysis was performed by Wilcoxon matching pairs test. Data are presented as mean and standard deviation.

## Results

The mean rate of sevoflurane infusion was 3.0 ± 0.6 ml/h, sevoflurane concentration - 0.5 ± 0.2%. We achieved the target sedation level (from -2 to -3 RASS) in all the patients. Significant fall of AAI index (AEP) was observed during the period of sedation (from 67.40 (12.5) to 42.4 (9.3) (p < 0.05). ANI rised during the period of sedation from 74.8 (6.8) to 96.0 (2.34), p < 0.05, and reflected adequate analgesia. MAP decreased during the therapy but remained in normal ranges. Increase of cardiac index (CI) and fall of system vascular resistance (SVR) (table) reflected more effective heart work that may be induced by cardioprotective effect of sevoflurane.

## Conclusions

Inhalational anaesthetic sevoflurane provides adequate level of sedation and analgesia during first 5 hours in patients in ICU after surgery and has no negative influence on circulation parameters.

**Table 1:** Influence of Sevoflurane on Haemodynamics

Parameters	Baseline	1 hour	2 hour	3 hour	4 hour	5 hour
MAP, mm Hg	90.4 (12,5)	89.0 (12,0)	85,9 (10.8)*	82.7 (7.5)*	81.6 (8.8)*	80.5 (10.9)*
CI, ml/min/m2	1.64 (0.71)	1.85 (0.74)	2.10 (0.68)*	2.20 (0,65)*	2.13 (0,11)*	2.23 (0.30)*
SVR, din·c·cm-5	3116.00 (950.15)	3097.75 (923.07)	3142.25 (940.86)	2920,75 (986.36)	2100,3* (130.42)	2387,6* (584.0)

* - significant comparing with baseline (p < 0,05).
